# Fabrication and properties evaluation of chitosan/BaTiO_3_ composite membranes for the periodontitis treatment

**DOI:** 10.1038/s41598-023-50929-0

**Published:** 2024-01-10

**Authors:** Aydin Houshyar, Mehdi Ahmadian, Yashar Azizian-Kalandaragh, Noushin Amirpour, Hossein Salehi

**Affiliations:** 1 Department of Materials Engineering, Isfahan University of Technology, Isfahan, 84156-83111 Iran; 2 Department of Physics, University of Mohaghegh Ardabili, Ardabil, 56199-13131 Iran; 3 Department of Photonics, Faculty of Applied Sciences, Gazi University, 06500 Ankara, Turkey; 4 Photonics Application and Research Center, Gazi University, 06500 Ankara, Turkey; 5 Department of Anatomical Sciences and Molecular Biology, School of Medicine, Isfahan University of Medical Sciences, Isfahan, 81746-73461 Iran

**Keywords:** Biotechnology, Materials science

## Abstract

Periodontitis gradually damages the hard and soft tissues surrounding the tooth, leading to tooth loss. In recent years, the use of biomaterials in periodontitis treatment has expanded, including gels, nanoparticles, microparticles, fibers, and membranes. Among these, membranes have more clinical applications. Due to the ability of the piezoelectric material to regenerate damaged tissues, the aim of this study was to create piezoelectric composite membranes. To achieve this, Barium titanate powder (BaTiO_3_ powder)—a piezoelectric substance—was synthesized using the hydrothermal method and analyzed with X-ray diffraction (XRD) and Field emission scanning electron microscopy (FESEM). Four types of membranes were fabricated using solvent casting method: three composite membranes with chitosan matrix and BaTiO_3_ fillers (at 3%, 6%, and 9% weight), and one chitosan membrane without BaTiO_3_. The microstructure of the membrane surfaces, agglomeration of BaTiO_3_ in membranes, and hydrophilicity, antibacterial, and electrical properties of the membrane were also investigated. The results indicated that membranes containing 3 and 6% BaTiO_3_ had suitable surface structure for the periodontitis treatment. Agglomeration of BaTiO_3_ particles was higher in the membrane containing 9% BaTiO_3._ The large amount of BaTiO_3_ improved the antibacterial properties of the membranes. Additionally, the membranes containing BaTiO_3_ had high electrical properties, especially those with 3% and 6% BaTiO_3_. Therefore, composite membranes containing BaTiO_3_, especially membranes containing 6% BaTiO_3_, are more favorable options than those without BaTiO_3_ for periodontitis treatment.

## Introduction

Periodontitis is the sixth chronic disease that affects more than 743 million people worldwide^1^. The periodontium consisting of gingiva, periodontal ligament, cementum, and alveolar bone that mechanically supports the teeth and plays an important role in transmitting the mechanical forces generated during chewing^2^. Periodontal disease is a bacterial infection resulting due to a complex interaction between microorganisms found in dental plaque and host immune response. The disease is characterized by inflammation and progressive destruction of periodontium components^3^. Among all bacteria, anaerobic Gram-negative bacteria play a more important role in forming periodontitis^3^. Gingivitis is an earliest stage of periodontal disease and can be prevented with thorough plaque removal and maintenance of effective oral hygiene^4^. The disease if left untreated leads to periodontitis with alveolar bone destruction and tooth loss^4,5^. There are many researches which showed that there is a special link between periodontitis and some systemic diseases^1,6,7^.

Today, among synthetic biomaterials, synthetic membranes known as GTR (Guided tissue regeneration) barrier membranes have found wide clinical use for periodontitis treatment^8–11^. These membranes are broadly divided into three generations^12^. The third-generation membranes are more useful than the first- and second-generation membranes for the treatment of periodontitis. These are biodegradable membranes which prevent unwanted cell penetration to the damaged site and contain antibacterial drugs, growth factor and or bioactive fillers^12^. Due to some limitations related to membranes containing drugs (large dose requirement)^13^ and growth factors (short half-life, large dose requirement, and high cost)^14^, it seems that the use of biodegradable membranes containing bioactive fillers are more useful and economical. Natural collagen polymers are one of the most common materials used in the fabrication of GTR membranes. The disadvantages of these polymers are the fast degradation rate and weak mechanical strength^15^. Aliphatic thermoplastic polymers such as PLA (polylactic acid), PGA (polyglycolic acid), and their copolymers are the most common synthetic degradable polymers. The disadvantages of these polymers are lack of cell adhesion and flexibility^5^, and the acid accumulation during the degradation of these polymers may significantly reduce the pH of the damaged site and lead to a chronic inflammatory response^11^.

Chitosan application in bone tissue engineering has increased due to the similarity of chitosan structure with the bone extracellular matrix structure and its promising biological properties such as biocompatibility, biodegradability, degradation products non-toxicity, and antimicrobial properties^5^. Unlike synthetic polymeric biomaterials such as PLA, which remains rigid in the aqueous environment, chitosan is flexible in the aqueous environment. The membrane flexibility is useful during the membrane placement in the damaged site, and it makes the membrane to be implanted optimally^5,15^. Also, chitosan has a good affinity with human periodontal ligament cells and facilitates osteoblasts adhesion and proliferation, which are responsible for new bone formation^5^.

In previous studies, bioactive fillers such as natural hydroxyapatite fillers extracted from chicken femur^5^, synthetic hydroxyapatite^16,17^, bioactive glasses^4,15^, multilayer carbon nanotubes^18^ and beta-tricalcium phosphate^19^ have been used in the composition of GTR composite membranes. The main purpose of using these fillers in membrane composition has been to improve the ability of apatite formation on the membrane surfaces. But apart from improving the ability of apatite formation, improving the cell activities on the membrane surface (adhesion and proliferation of periodontium’s cells) also increases the efficiency of the membrane in periodontitis treatment^5^. Hence, in the chemical composition of GTR membrane, we need some kind of fillers that simultaneously increase the ability of apatite formation and the adhesion and proliferation of periodontium’s cells on the membrane surface. Generated electrical pulses of piezoelectric scaffolds improve specific cellular activities such as cell adhesion, migration, proliferation, and differentiation^20^. Electrical effects including piezoelectricity, pyroelectricity, and dielectricity play an important role in bone regeneration^21^. BaTiO_3_ has interesting ferroelectric, piezoelectric, pyroelectric properties with high dielectric constant^22^, and bioactivity^20^. It has also shown antibacterial activity against both gram-positive and gram-negative bacteria^23,24^.

Considering the favorable properties mentioned about chitosan and BaTiO_3_, the aim of our study is to fabricate suitable composite membranes with chitosan matrix and BaTiO_3_ fillers use for the treatment of periodontitis.

## Materials and methods

### Materials

In this research, Titanium oxide (TiO_2_, Loba Chemie, 25nm), Sodium hydroxide (NaOH, Merck), Barium hydroxide (Ba(OH)_2_, Merck), 1- Decanethiol (As a surfactant, Aldrich), Chitosan powder (medium molecular weight, 190–310 KDa, 75–85% deacetylated, Aldrich), Phosphate-buffered saline (PBS, Nanomer Biomaterials Institute), Acetic acid (Aldrich), Mueller–Hinton agar (QUELAB), and Normal saline (Iranian Parenteral and Pharmaceutical Company) were used.

### Synthesis of BaTiO_3_ powder

BaTiO_3_ powder was synthesized using the hydrothermal method^25^. First, a solution containing 0.240 g (0.0030 mol) TiO_2_ in 21 mL distilled water and a solution containing 0.945 g (0.0055 mol) Ba(OH)_2_ in 21 mL distilled water were prepared. While stirring both solutions in 2 separate beakers, one of the solutions was added drop by drop to the other solution to form a new solution. A solution containing 1.200 g NaOH (0.0300 mol) in 21 mL distilled water was prepared and added drop by drop to the new solution while stirring. Finally 450 µL 1-decanethiol was added to the new solution until the final solution is prepared (It should be noted that the stirring process in all stages was accomplished by ultrasonic bath for 15 min). The final solution was transferred to the autoclave and the autoclave was placed inside the oven under 180 °C for 2 h. After 2 h, the product inside the autoclave was washed five times with distilled water, and dried at room temperature. To achieve the final product, the dried powder was placed in the furnace at 800 °C for 2 h^25^.

### Membrane preparation

Four membranes with chitosan matrix and BaTiO_3_ fillers (with weight percentages of 0, 3, 6, and 9) were fabricated by solvent casting method. For fabricating a chitosan membrane without BaTiO_3_, First, 0.5 g chitosan was poured into a beaker containing 25 mL diluted acetic acid (24.5 mL distilled water plus 0.5 mL pure acetic acid), and the mixture was stirred (500 rpm) by a magnetic stirrer at room temperature for 5 h until chitosan powder is completely dissolved. Then, the solution containing chitosan was poured into a petri dish (with a diameter of 10 cm) and placed in an oven at 40 °C for 48 h to remove the solvent from the petri dish. In order to neutralize the membrane inside the petri dish, NaOH solution (7 g NaOH in 100 mL distilled water) was placed inside the petri dish for 15 min. After removing the NaOH solution, the membrane was separated from the Petri dish and washed 5 times with distilled water. Finally, the membrane was placed in an incubator at 37 °C for drying. For fabricating composite membranes containing BaTiO_3_, 0.015 g (3 wt %), 0.030 g (6 wt %), and 0.045 g (9 wt %) BaTiO_3_ were poured into three separate beakers containing 24.5 mL distilled water and were ultrasonicated (by an Ultrasonic Homogenizer, FAPAN 1200UT) for 5 min. Next, 0.5 mL of acetic acid was added to each beaker. Then, 0.485 g, 0.470 g, and 0.455 g chitosan powder were added to the beakers containing 0.015 g, 0.030 g, and 0.045 g BaTiO_3_, respectively, and the materials inside the beakers were stirred (500 rpm) with the magnetic stirrer at room temperature for 5h until chitosan powder is completely dissolved. The contents of 3 beakers were poured into 3 separate Petri dishes and placed in the oven at 40 °C for 48 h to remove the solvent from the Petri dishes. The steps of neutralization, washing, and drying of these membranes were accomplished in the same way as the chitosan membrane without BaTiO_3_^5^.

**Figure 1:**
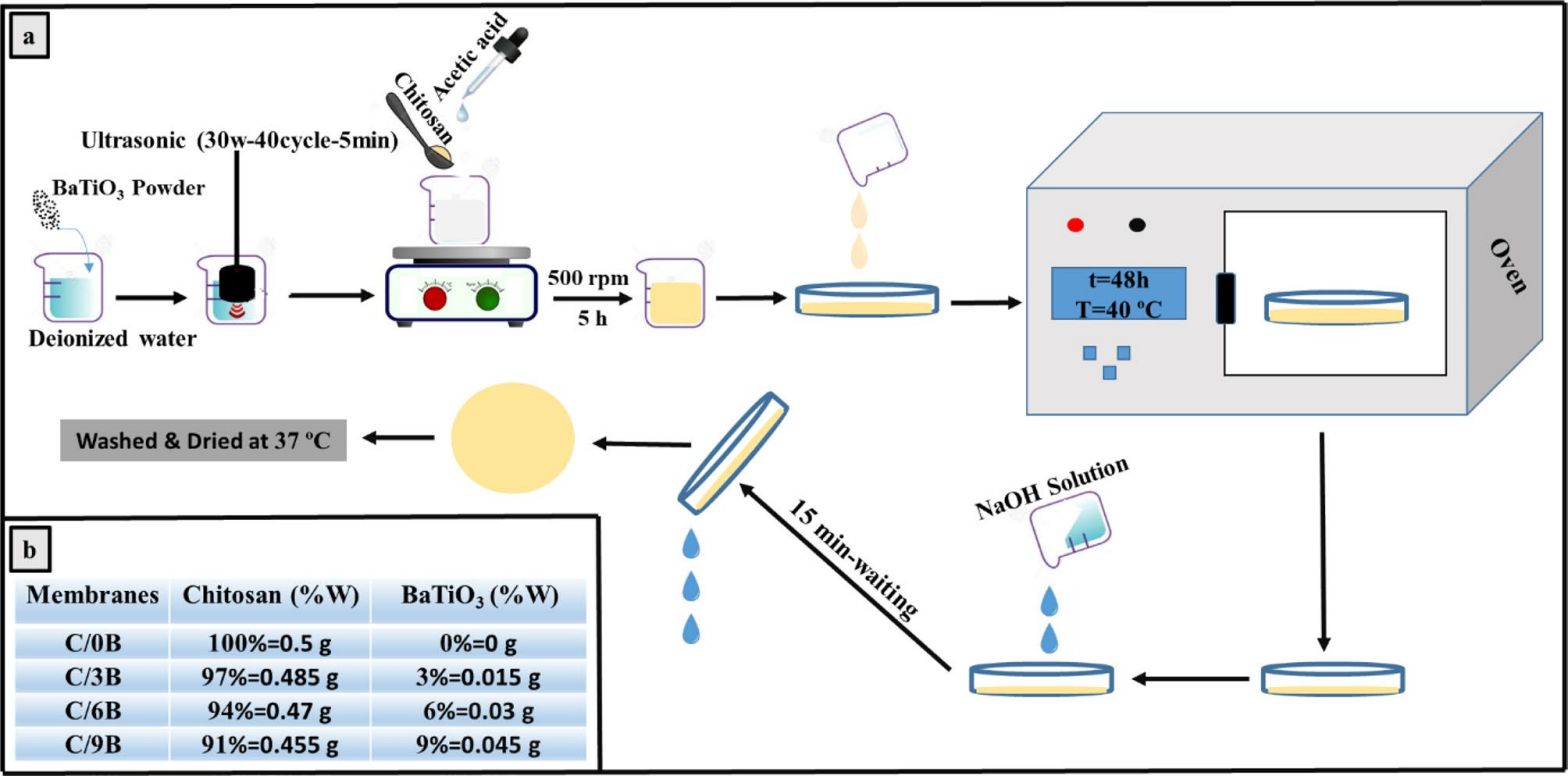
Fabrication method of membranes containing BaTiO_3_ (**a**) and weight percentages of membranes constituents (**b**).

After fabricating the membranes, membranes containing 0, 3, 6, and 9 wt. % BaTiO_3_ were coded as C/0B, C/3B, C/6B and C/9B, respectively. Figure 1a and b show the fabrication method of membranes containing BaTiO_3_ and the weight percentages of the membranes ‘constituents, respectively.

Membranes in the final stage of their fabrication (when they are separated from the Petri dish) will have two surfaces with different characteristics. A surface of the membrane that is in contact with the air (SCA surface) will be rougher than the other surface of the membrane that is in contact with the petri dish (SCP surface)^5^.

### Phase identification in the synthesized powder and the membranes surfaces

X-ray diffractometer (Philips Netherlands Company with Cukα radiation, wavelength 1.54 A°) was used for phase analysis of the synthesized powder and the SCA surfaces of the membranes^26^.

### Imaging of the synthesized powder and the membranes surfaces

After the gold coating on the powder, a field emission electron microscope (FESEM, TESCAN MIRA-3) was used to capture an image of the powder. Samples with dimensions of 1 × 1 cm^2^ were prepared from the fabricated membranes and gold coating was applied on the SCA and SCP surfaces of the membranes. Then, the SCA and SCP surfaces were imaged by the field emission electron microscope, and the distribution of barium, titanium and oxygen elements on the SCA surfaces of membranes was surveyed by mapping system^15^.

**Figure 2:**
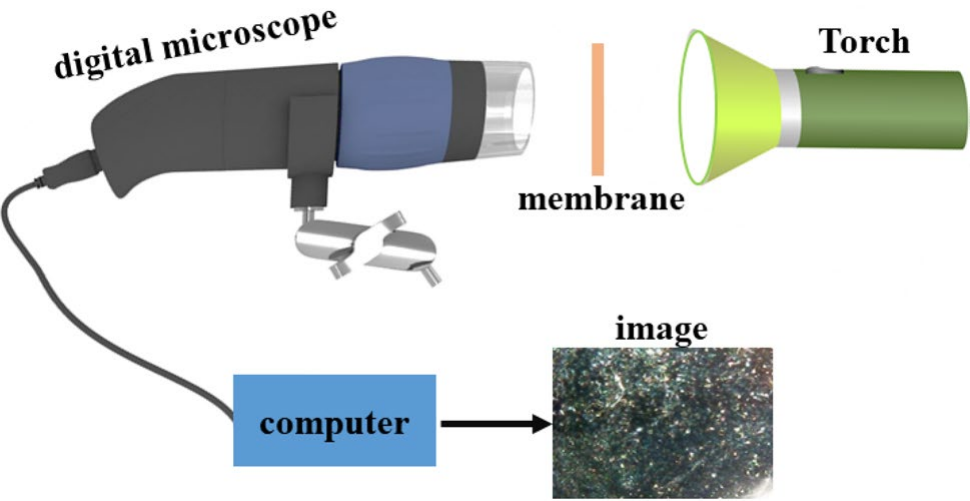
Imaging schematic of the membranes surfaces.

Prepared circular samples of membranes with 1cm diameter were placed on an opaque black surface and their surfaces were photographed by a digital camera. Then, the circular samples surfaces were imaged by a digital microscope (with the same magnification for all samples). Figure 2 illustrates imaging method of membranes surfaces by the digital microscope^27^.

### Ultraviolet–visible spectroscopy for membranes

Membranes with dimensions of 1 × 5 cm^2^ were prepared. Then, the wavelength-absorbance curves of the membranes were obtained by a visible-ultraviolet spectrometer (Shimadzu UV-1800, the wavelength range: 200–800 nm)^28^.

### Hydrophilicity assay for membranes

Membranes hydrophilicity was evaluated by the sessile drop method. In order to perform this test, membranes with dimensions of 1 × 1 cm^2^ were prepared (5 samples from each membrane) and both surfaces of the membranes (SCA and SCP surfaces) were positioned 3 mm away from the tip of a Hamilton syringe filled with 5 µL of PBS drop. Then, PBS drops were placed on the membrane surfaces, and images of the drops were captured using a digital camera. This procedure was repeated five times for each surface of C/0B, C/3B, C/6B and C/9B membranes. Finally, ImageJ software was used to measure the contact angle of the PBS drops on the membrane surfaces^29^.

### Antibacterial assay for membranes (agar diffusion method)

In this test, Escherichia coli bacteria (an anaerobic Gram-negative bacterium) was used^23,30^. To prepare the bacterial medium, first, 4 to 5 colonies of pure-young standard strain of Escherichia coli bacterium were transferred to normal saline solution and the bacteria concentration in normal saline solution was standardized using a 0.5 McFarland standard. Next, 10 µL of bacterial suspension (containing 1.5 × 10^8^ bacteria) was lawn-cultured on a plate containing Mueller–Hinton agar using a sterile swab, and the plate was placed at room temperature for 10 min to absorb its moisture. Then, C/0B, C/3B, C/6B, and C/9B membranes were punched in a circle shape (diameter 6 mm) and sterilized by a UV device. The membranes finally were placed on the plate surface and the plate was placed upside down for 24 h at 37 °C inside the incubator. After 24 h, the zone of inhibition around the membranes were measured by using vernier calliper in mm and photographed by a digital camera^31^.

### Electrical test for membranes

**Figure 3:**
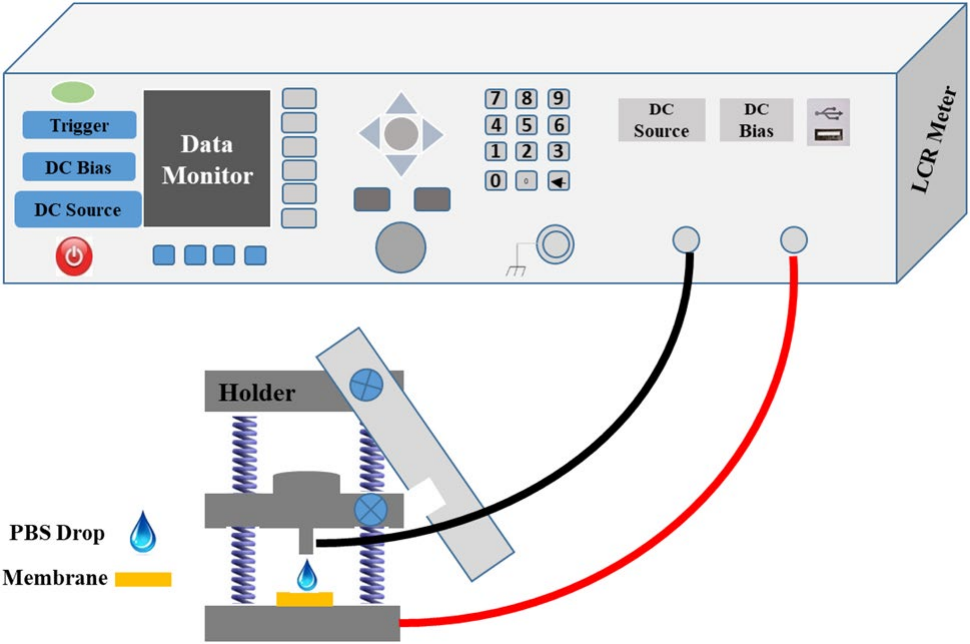
Electrical test schematic for C/0B, C/3B, C/6B, and C/9B membranes.

Samples with dimensions of 1 × 1 cm^2^ were prepared from C/0B, C/3B, C/6B, and C/9B membranes. The samples were individually placed on the anvil of holder connected to the LCR Meter (KEYSIGHT, E4980AL) (see Fig. 3). Then 4 µL of PBS solution was poured on the membrane surfaces and the holder rod was placed on the PBS drop. After 1 min, the electrical parameters of each membrane, including electrical conductivity (G) and electrical capacity (C) were recorded by the LCR Meter device at different frequencies (f: 10^2^–10^3^ Hz). The dielectric constant (ε_∘_) and intrinsic conductivity ($\sigma$) of the membranes were calculated by following formulas:

1 $$\varepsilon {\prime}=(C\times d)/({\varepsilon }_{\circ }\times A),$$

2 $$\sigma =(G\times d)/A.$$

In these formulas, ɛ˳, A, and d are the vacuum permeability constant, the effective area of the membrane (area of the membrane that is in contact with the cross section of the holder rod), and the membrane thickness, respectively^32^.

## Results and discussion

### The structure and purity of the synthesized powder

**Figure 4:**
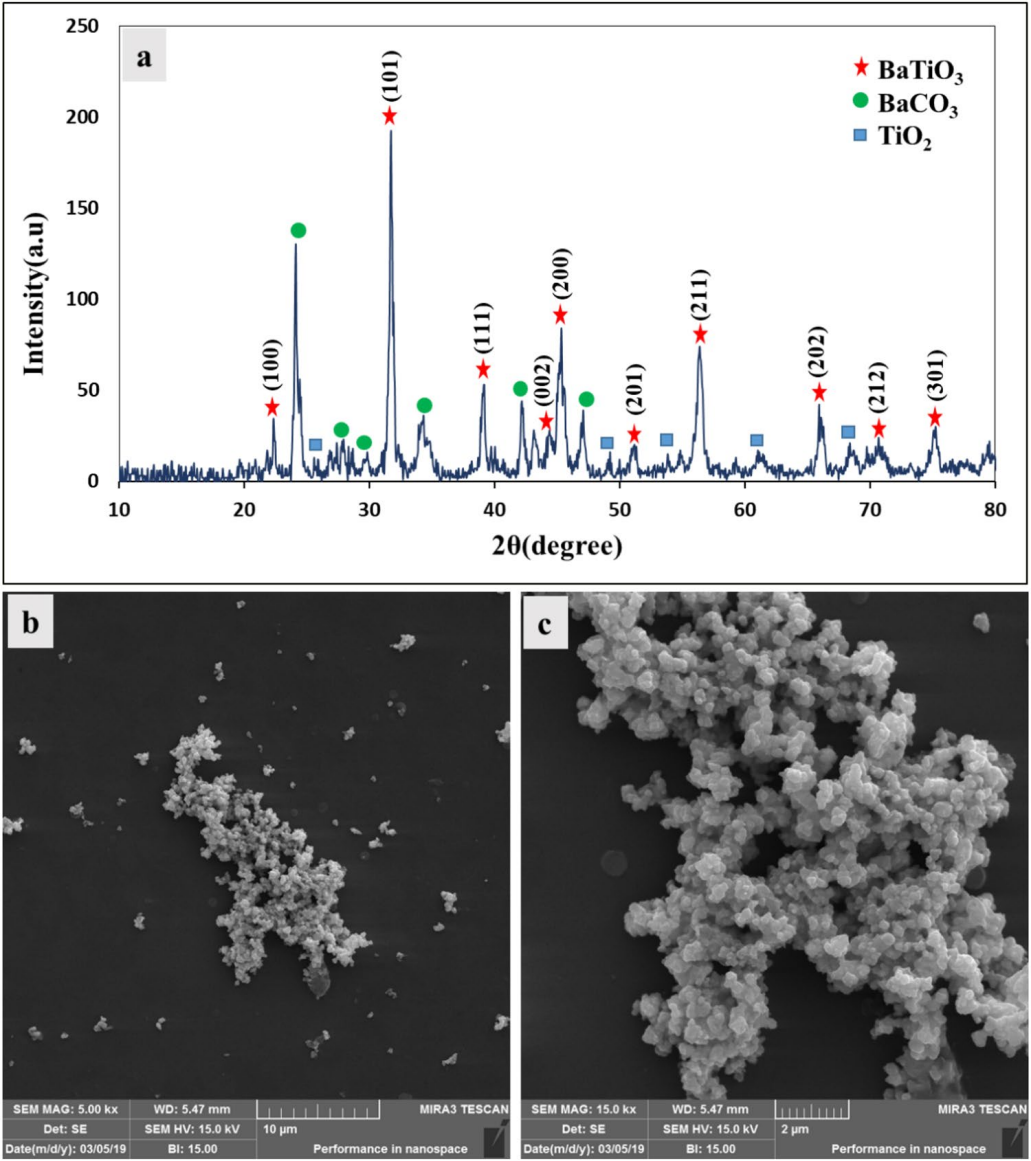
X-ray diffraction pattern (**a**) and FESEM images of BaTiO_3_ particles (**b**,**c**).

Figure 4a shows the X-ray diffraction pattern of the synthesized powder. The nature of the powder diffraction pattern was investigated using High Score Plus software. According to software search, individual peaks at angles of 22.3°, 31.7°, 39°, 45.2°, 45.5°, 51.1°, 56.4°, 66°, 70.7° and 75.3° are related to crystal planes of (100), (101), (111), (002), (200), (201), (211), (202), (212) and (301) of tetragonal BaTiO_3_, respectively. The appearance of two separate peaks belonging to the crystal planes of (002) and (200) at the position of 2θ ~ 45° indicates the formation of tetragonal BaTiO_3_^33,34^. Also, according to software search, other peaks appearing in this pattern are related to barium carbonate and titanium dioxide. One of the common impurities in the BaTiO_3_ nanostructure preparation (by different methods) is the presence of barium carbonate, which is not removed from the final product even after washing and purification^35^. Figure 4b and c show FESEM images of the synthesized BaTiO_3_ powder. Using ImageJ software, the average size of BaTiO_3_ particles in Fig. 4c was estimated to be approximately 120 nm.

### Investigating the microstructure of membrane surfaces

**Figure 5:**
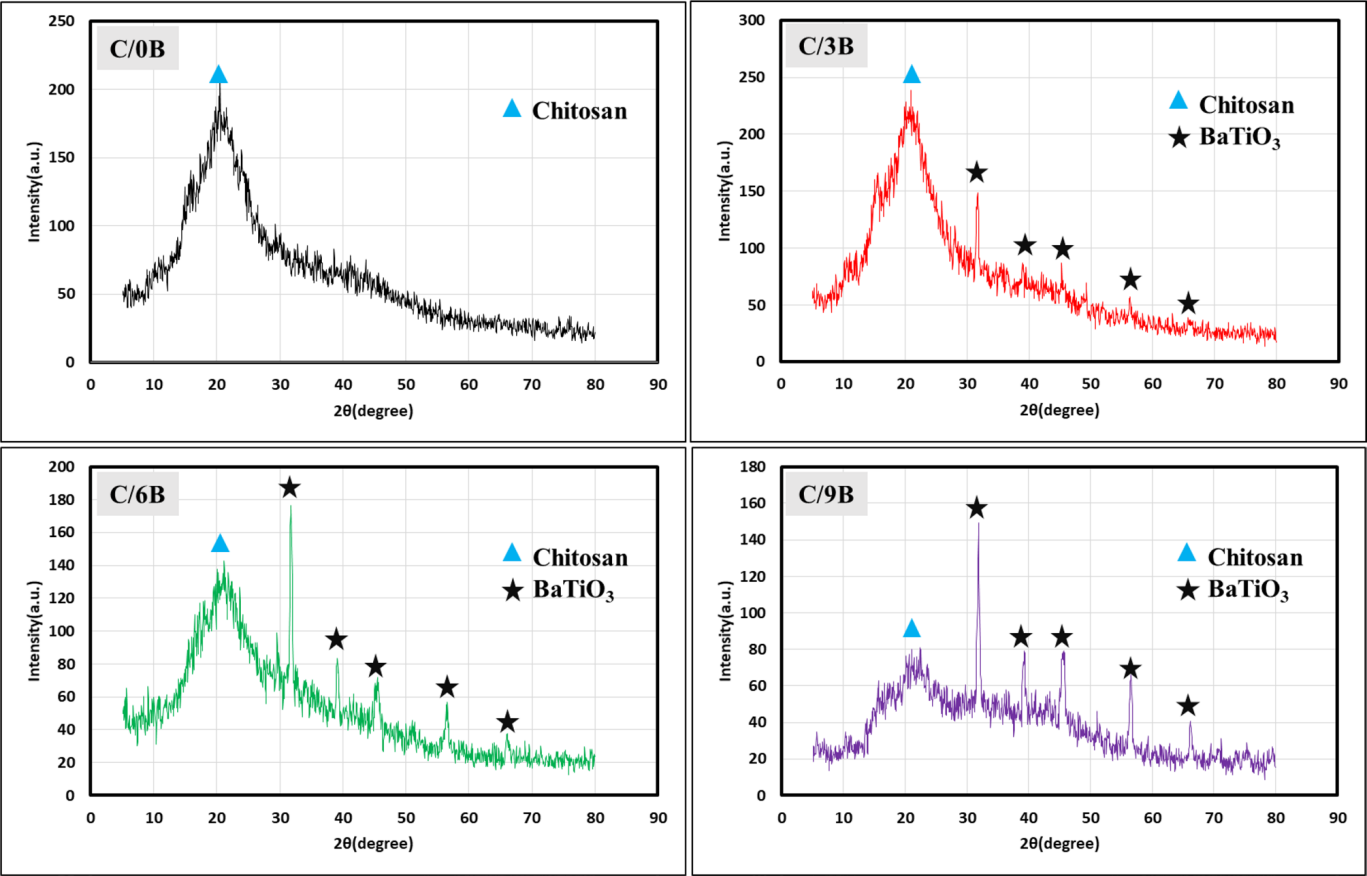
X-ray diffraction pattern for the SCA surfaces of C/0B, C/3B, C/6B, and C/9B membranes.

Figure 5 shows the X-ray diffraction pattern of the SCA surfaces of C/0B, C/3B, C/6B, and C/9B membranes. By comparing Figs. 4a and 5, it can be observed that there are some peaks related to BaTiO_3_ powder in the membranes containing BaTiO_3_ (especially the most intense peak at the angle of 31.7°). In C/6B and C/9B membranes, the peaks related to BaTiO_3_ are more visible, which is due to the high amount of BaTiO_3_ in their SCA surfaces. Also, a peak is observed in all membranes at angle of 20° which is related to chitosan^36,37^. By comparing Figs. 4a and 5, the removal of barium carbonate impurity peaks in the membranes containing BaTiO_3_ seems certain. The removal of these peaks was predictable because in the early stages of membrane fabrication, a mixture of acetic acid and distilled water (24.5 mL plus 0.5 mL acetic acid) was used to dissolve chitosan, and barium carbonate can be dissolved even in diluted acetic acid^38^.

**Figure 6:**
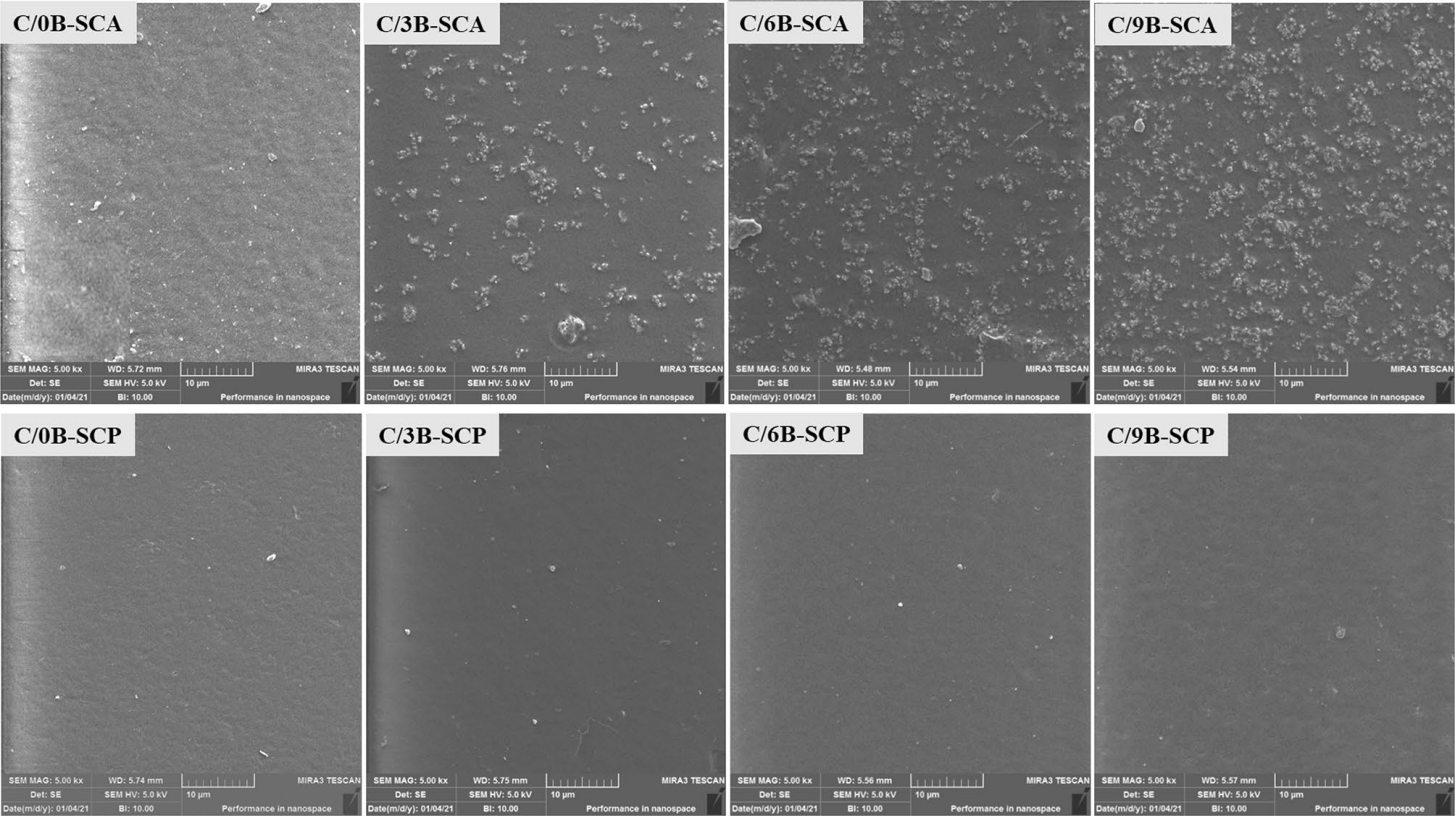
FESEM images of the SCP and SCA surfaces in C/0B, C/3B, C/6B, and C/9B membranes.

Figure 6 shows FESEM images of the SCA and SCP surfaces in C/0B, C/3B, C/6B and C/9B membranes. In this figure the presence of BaTiO_3_ particles on the SCA surfaces of membranes containing BaTiO_3_ is evident, and the amount of BaTiO_3_ on the SCA surfaces of C/3B, C/6B and C/9B membranes have increased, respectively. Also, there is not much difference between the SCA and SCP surfaces of C/0B membrane and both surfaces are smooth, while the SCA and SCP surfaces of membranes containing BaTiO_3_ (C/3B, C/6B and C/9B) are rough and smooth, respectively. Hence, it can be concluded that C/3B, C/6B, and C/9B membranes can be more suitable options for periodontitis treatment than C/0B membrane. Because the smooth surfaces of these membranes will be in contact with the gingival tissues, these surfaces prevent the proliferation and penetration of fibroblasts and epithelial cells into the damaged site (these cells inhibit periodontium formation). The rough surfaces of these membranes will be in contact with the damaged site so that these surfaces increase adhesion and proliferation of periodontium's cells that are responsible of periodontium repair^5^.

In general, the properties of composite membrane depend on the type, amount, and particle size of fillers. It also depends on distribution of fillers in the polymer matrix and the agglomeration of fillers^39^. Thus, all these factors together determine the properties of the composite membrane.

**Figure 7:**
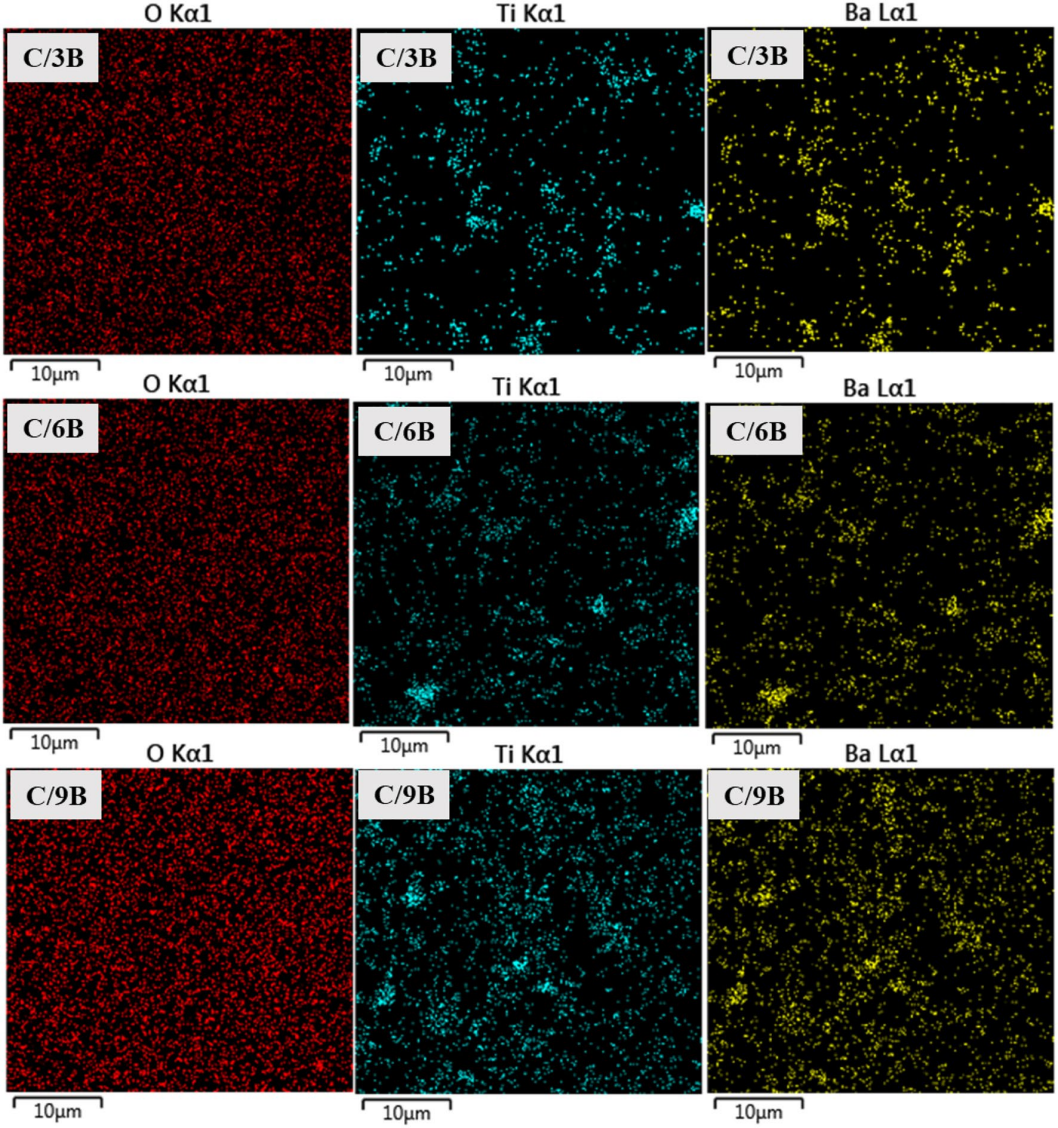
Elemental mapping: distribution of oxygen, barium, and titanium elements on the SCA surfaces of C/3B, C/6B, and C/9B membranes.

The distribution of BaTiO_3_ particles in the composite membranes (C/3B, C/6B, and C/9B membranes) can be evaluated by the distribution of the BaTiO_3_ constituent elements on the SCA surfaces of the membranes. Figure 7 presents the distribution of barium, titanium, and oxygen elements on the SCA surfaces of composite membranes by elemental mapping. It is possible to evaluate the distribution of BaTiO_3_ particles in the membranes by the distribution of titanium and barium elements in these membranes, whereas it is not possible to evaluate the distribution of BaTiO_3_ particles in the membranes by distribution of oxygen elements in the membranes because both chitosan (as the membranes matrix) and BaTiO_3_ (as the membranes filler) contain oxygen elements. In fact, due to the high oxygen element congestion on the SCA surfaces of the membranes, no difference is observed in the amount of oxygen element congestion on the SCA surfaces of C/3B, C/6B, and C/9B membranes. However, the congestion of titanium and barium elements in the C/3B, C/6B, and C/9B membranes have increased, respectively. The higher congestion of titanium and barium elements on the SCA surface of the C/9B membrane indicates the higher congestion of BaTiO_3_ particles in C/9B membrane, which can increase the agglomeration rate of BaTiO_3_ particles in this membrane.

In general, Figs. 6 and 7 demonstrate that the C/9B membrane has more filler congestion than the C/3B and C/6B membranes, which increases the high agglomeration possibility of fillers in the C/9B membrane. Filler agglomeration is one of the basic and controversial problems in the nanocomposites structure, which causes their properties reduction^4,40^. For example, in nanocomposite membranes, increasing the agglomeration of fillers can cause a decrease in mechanical properties^4^, dielectric properties^22^, hydrophilicity, and cell adhesion^26^.

**Figure 8:**
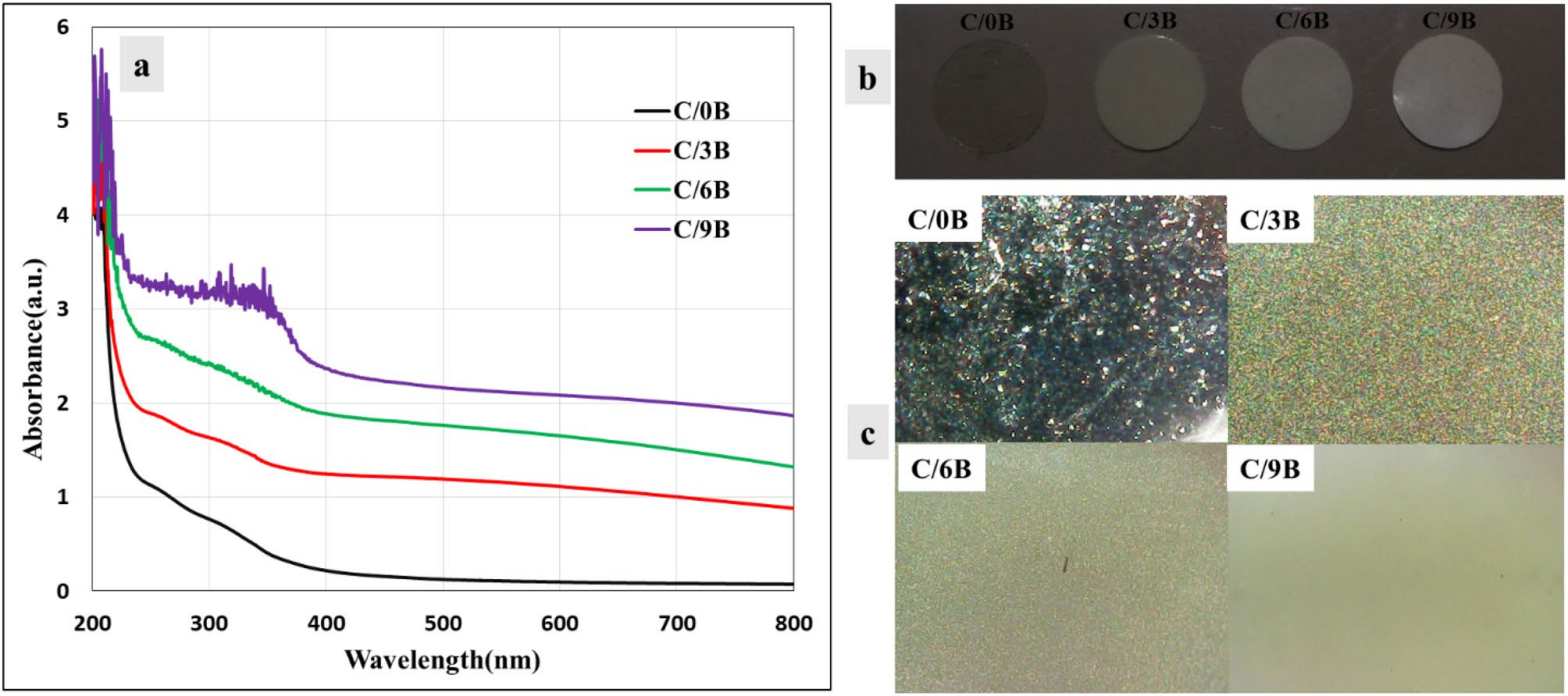
Absorbance–wavelength curves (**a**), circular samples (**b**) and microscopic images of C/0B, C/3B, C/6B, and C/9B membranes (**c**).

Figure 8a and c respectively shows the absorbance–wavelength curves of the membranes and the microscopic images of membrane surfaces (by the digital microscope), which were used for investigating the agglomeration of BaTiO_3_ particles prepared in this work (see Fig. 8b). It has been reported that increasing the filler agglomeration in composite films causes noise formation in the absorbance–wavelength or transmission-wavelength curves of the films^41^. Figure 8a also shows noise creation in a region of the absorbance–wavelength curve of the C/9B membrane (wavelength between 400 and 200 nm). Increasing the filler agglomeration has a great effect on reducing the transparency of the composite membrane^42^. In nanocomposite membranes, the increase in the filler agglomeration causes an increase in light scattering, which consequences can the reduction of membrane transparency and the foggy area formation (haze) on the membrane surfaces^43^. Figure 8c also shows the foggy area formation on the C/9B membrane surface. Thus, according to our results, Fig. 8a and c estimate high agglomeration of BaTiO_3_ fillers in the C/9B membrane.

### Investigating the membrane’s hydrophilicity

**Figure 9:**
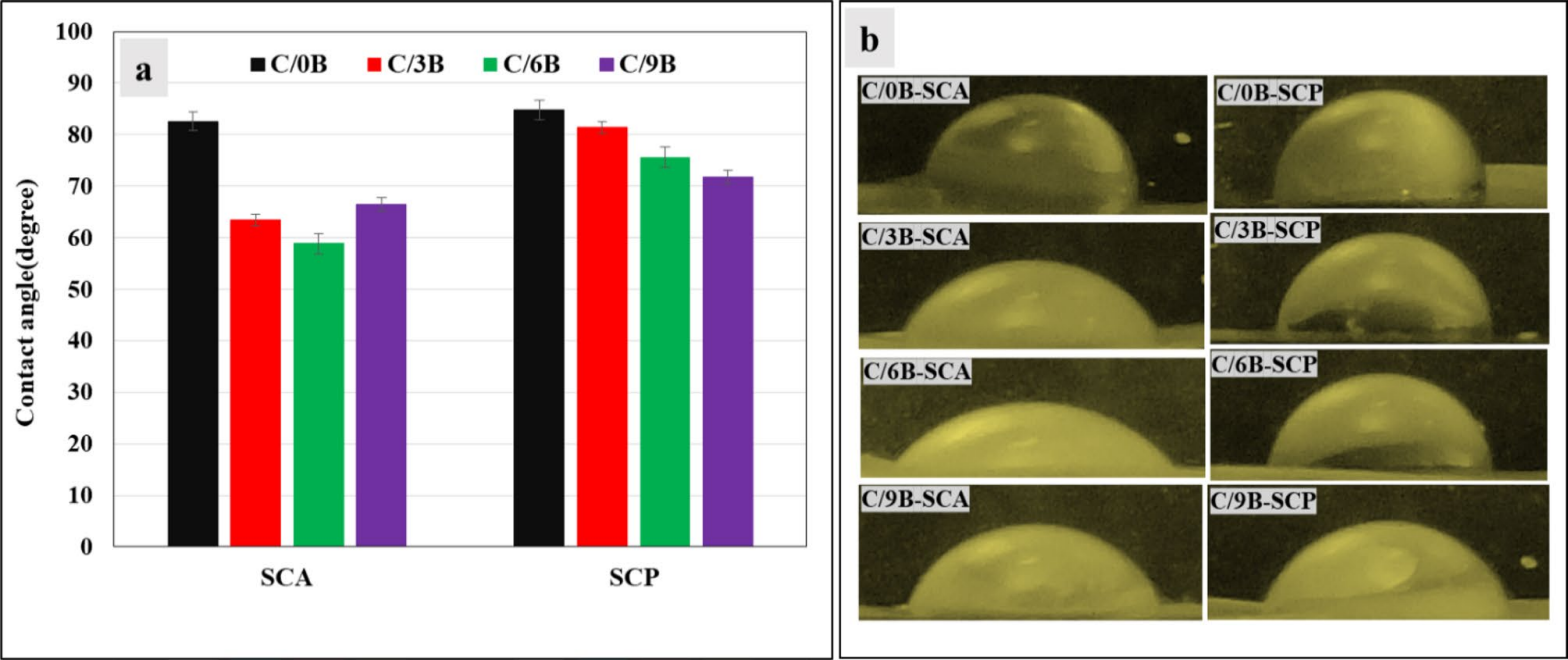
The PBS drops contact angles with the SCA and SCP surfaces (**a**) and PBS drops morphology on the SCA and SCP surfaces of C/0B, C/3B, C/6B, and C/9B membranes (**b**).

Figure 9a and b show graph of the contact angle and PBS drops morphology on SCA and SCP surfaces, respectively. In each of the C/0B, C/3B, C/6B, and C/9B membranes, the SCA surface is more hydrophilic than the SCP surface. This phenomenon is clearly observed in C/6B and C/3B membranes. But in C/9B and C/0B membranes, a difference of hydrophilicity between the SCA and SCP surfaces is very low. Hence, C/6B and C/3B membranes are more suitable than C/9B and C/0B membranes for periodontitis treatment because more hydrophilic surfaces of these membranes promote the proliferation of periodontium cells in to the damaged site and less hydrophilic surfaces prevents the penetration of fibroblasts in to the damaged site^4,15^.

### Investigating the membrane’s antibacterial properties

**Figure 10:**
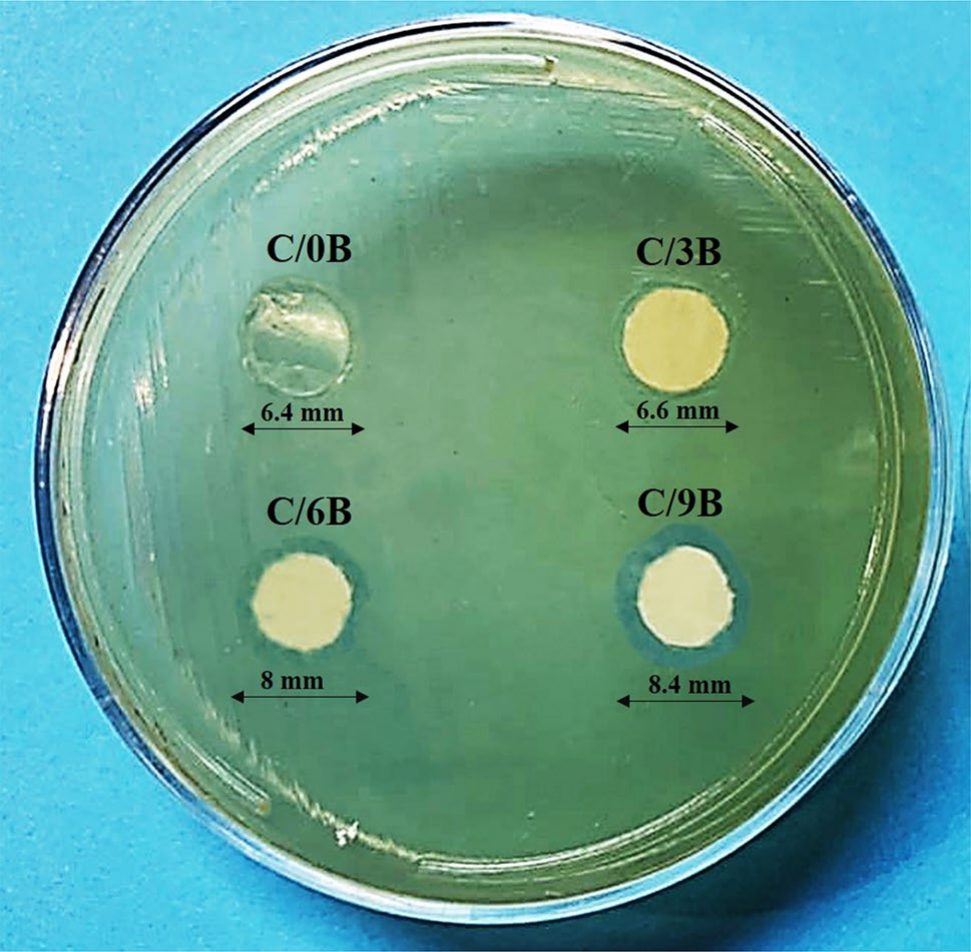
Zone of inhibition around the C/0B, C/3B, C/6B, and C/9B membranes after 24 h incubation in the bacterial medium (Escherichia coli bacteria).

Figure 10 shows the zone of inhibition around the membranes by using agar diffusion method. After 24 h of placing the membranes in the bacterial medium, the diameter of the bacterial inhibition zone in C/0B, C/3B, C/6B, and C/9B membranes increased to 6.4 mm, 6.6 mm, 8 mm, and 8.4 mm, respectively. Studies have shown that the antibacterial properties of BaTiO_3_ particles are directly related to the concentration of these particles^23,24^. Hence, it seems that the increase of BaTiO_3_ in the membranes causes an increase in the bacterial inhibition zone. In general, BaTiO_3_ in small amount (3 wt %) and chitosan polymer have a very small effect in improving the antibacterial properties of the membranes. While BaTiO_3_ in large amounts (6 and 9 wt %) has a relatively large effect on improving the antibacterial properties of the membranes.

### Investigating the membrane’s electrical properties

**Figure 11:**
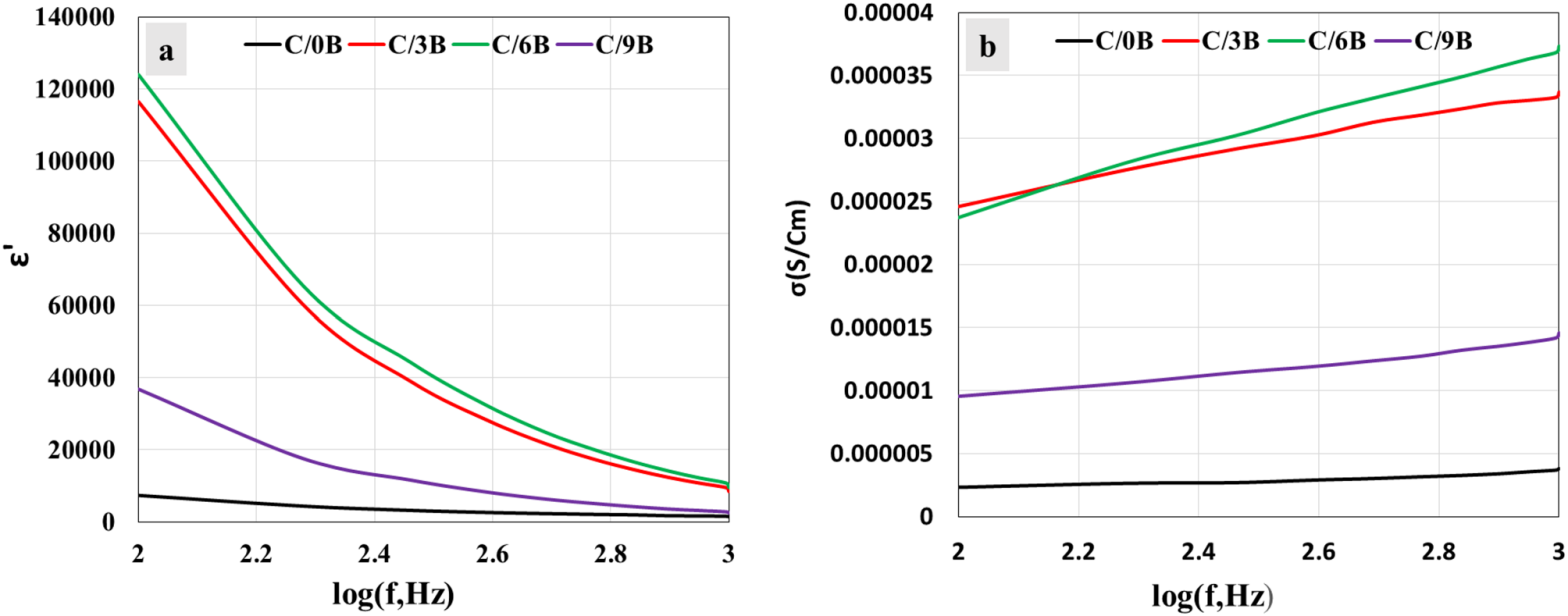
Dielectric constant-frequency curves (**a**) and intrinsic conductivity- frequency curves of C/0B, C/3B, C/6B, and C/9B membranes (**b**).

Figure 11 indicates that the electrical ability of membranes containing BaTiO_3_, especially C/3B and C/6B membranes, is more suitable than that of C/0B membrane for periodontium repair. When the membranes are placed on the damaged periodontium and then the alternating electric current applied to the damaged region, the dielectric constant and the intrinsic conductivity of the membranes will play important roles in the periodontium repair^44^. High intrinsic conductivity of the membranes enhances adhesion and proliferation of periodontium's cells that are responsible for periodontium repair^45,46^, while the high dielectric constant of the membranes promotes the ability of apatite formation on the membranes surfaces that is useful for repairing the bony component of periodontium^47,48^.

Figure 11a and b show the curves of dielectric constant and intrinsic conductivity of C/0B, C/3B, C/6B, and C/9B membranes in the frequency range of 10^2^–10^3^ Hz, respectively. At any frequency, the dielectric constant and intrinsic conductivity of the membranes containing BaTiO_3_, especially C/3B and C/6B membranes, are more than those of the membranes without BaTiO_3_ (C/0B). Also, at any frequency, the dielectric constant and intrinsic conductivity of the C/9B membrane are less than those of the C/3B and C/6B membranes, which is probably due to the high agglomeration of BaTiO_3_ particles in C/9B membrane^22^.

## Conclusion

In this study, the powder of BaTiO_3_ nanoparticles is successfully synthesized by hydrothermal method. Using the solvent casting method, it is possible to fabricate suitable chitosan membranes containing 0, 3, 6, and 9 wt % BaTiO_3_.

In our study, XRD analysis indicates that the chemical composition of the composite membranes containing BaTiO_3_ does not contain barium carbonate impurity, which usually present in the BaTiO_3_ powder. The membranes containing 3 and 6% BaTiO_3_ have a rough surface with high hydrophilicity and a smooth surface with low hydrophilicity, making them superior for use in periodontitis treatment because the rough surface with high hydrophilicity can be in contact with the damaged periodontium, promoting adhesion and proliferation of periodontium’s cells, leading to faster repair of the damaged tissue. The smooth surface with low hydrophilicity can be in contact with the gingival tissue and prevent unwanted cell penetration to the damaged site. The composite membrane containing 9% BaTiO_3_ has a high particle congestion rate, which increases the agglomeration of BaTiO3 particles in the membrane. The large amount of BaTiO_3_ improves the antibacterial properties of the membranes. Furthermore, the electrical ability of membranes containing BaTiO_3_, especially those containing 3% and 6% BaTiO_3_, is more than that of membranes without BaTiO_3_ which plays an important role periodontium repair.

In conclusion, this study demonstrates that composite membranes containing BaTiO_3_, especially membranes containing 6% BaTiO_3_, are more suitable for periodontitis treatment than chitosan membranes without BaTiO_3_.

## Data Availability

The datasets used and/or analyzed during the current study are available from the corresponding author upon reasonable request.
